# Attenuation of Doxorubicin‐Induced Small Intestinal Mucositis by Pectins is Dependent on Pectin's Methyl‐Ester Number and Distribution

**DOI:** 10.1002/mnfr.202100222

**Published:** 2021-08-07

**Authors:** Martin Beukema, Éva Jermendi, Taco Koster, Kohji Kitaguchi, Bart J. de Haan, Marco Alexander van den Berg, Marijke M. Faas, Henk A. Schols, Paul de Vos

**Affiliations:** ^1^ Department of Pathology, Medical Biology and Immunoendocrinology, Division of Medical Biology University Medical Center Groningen Hanzeplein 1, 9713 GZ Groningen The Netherlands; ^2^ Laboratory of Food Chemistry Wageningen University Bornse Weilanden 9, Wageningen WG 6708 The Netherlands; ^3^ Department of Applied Life Science, Faculty of Applied Biological Sciences Gifu University 1‐1 Yanagido Gifu City 501‐1193 Japan; ^4^ DSM Biotechnology Center Alexander Fleminglaan 1 Delft AX 2613 The Netherlands

**Keywords:** chemotherapy, degree of blockiness, degree of methyl‐esterification, doxorubicin, mucositis, pectin

## Abstract

**Scope:**

Intestinal mucositis is a common side effect of the chemotherapeutic agent doxorubicin, which is characterized by severe Toll‐like receptor (TLR) 2‐mediated inflammation. The dietary fiber pectin is shown to prevent this intestinal inflammation through direct inhibition of TLR2 in a microbiota‐independent manner. Recent in vitro studies show that inhibition of TLR2 is determined by the number and distribution of methyl‐esters of pectins. Therefore, it is hypothesized that the degree of methyl‐esterification (DM) and the degree of blockiness (DB) of pectins determine attenuating efficacy on doxorubicin‐induced intestinal mucositis.

**Methods and Results:**

Four structurally different pectins that differed in DM and DB are tested on inhibitory effects on murine TLR2 in vitro, and on doxorubicin‐induced intestinal mucositis in mice. These data demonstrate that low DM pectins or intermediate DM pectins with high DB have the strongest inhibitory impact on murine TLR2‐1 and the strongest attenuating effect on TLR2‐induced apoptosis and peritonitis. Intermediate DM pectin with a low DB is, however, also effective in preventing the induction of doxorubicin‐induced intestinal damage.

**Conclusion:**

These pectin structures with stronger TLR2‐inhibiting properties may prevent the development of doxorubicin‐induced intestinal damage in patients undergoing chemotherapeutic treatment with doxorubicin.

## Graphical abstract

Pectins with a low degree of methyl‐esterification (DM) or intermediate DM pectins with a high degree of blockiness have strong Toll‐like receptor (TLR) 2 inhibiting properties and prevent the induction of doxorubicin‐induced intestinal mucositis. Through inhibition of TLR2, these pectins prevent induction of doxorubicin‐induced apoptosis. A lower level of apoptosis together with barrier‐enhancing properties of pectins prevents bacterial translocation and subsequent intestinal damage.

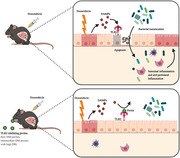

## 1 Introduction

Intestinal mucositis is a common side effect of the chemotherapeutic agent doxorubicin, which is characterized by severe inflammation and ulceration of the intestinal lining.^[^ ^1^ ^]^ Doxorubicin induces apoptosis of cancer cells by intercalating into the DNA,^[^ ^2^ ^]^ but doxorubicin also induces apoptosis of fast dividing cells in the intestine with mucositis as a consequence. Mucositis can cause severe discomfort in patients^[^ ^1^ ^]^ and that can be dose‐limiting or prevent completion of the chemotherapeutic treatment.^[^ ^3^, ^4^ ^]^ Anti‐inflammatory agents have been used to suppress doxorubicin‐induced intestinal inflammation, but there is no effective treatment available besides stopping the chemotherapeutic treatment with severe consequences for progression of disease.^[^ ^5^ ^]^ New therapeutic strategies limiting the side effect of doxorubicin on the intestinal lining are urgently needed.

Doxorubicin‐induced intestinal mucositis is characterized by a fast induction of apoptosis in the stem cell regions in crypts of the small intestine,^[^ ^6^ ^]^ followed by mucosal damage that includes immune cell infiltration, villus, and crypt degeneration.^[^ ^7^, ^8^ ^]^ The apoptosis of epithelial cells increases intestinal permeability allowing bacterial translocation to the underlying tissues.^[^ ^9^ ^]^ Consequently, more apoptosis of the epithelium and ulceration might occur. Repair of intestinal cells occurs in the final phase in which the crypt length increases and the mucosal lining returns to its original morphology.^[^ ^10^ ^]^ In addition to the inflammation of the intestinal mucosal, intestinal mucositis is also characterized by inflammation in the peritoneal cavity.^[^ ^11^ ^]^

Recent studies have demonstrated that the development of intestinal mucositis was dependent on Toll‐like receptor (TLR) activation.^[^ ^7^, ^11^, ^12^ ^]^ TLRs are pattern recognition receptors that are expressed on intestinal epithelial cells and immune cells. They are activated after recognition of microbial derived pathogen associated molecular patterns (PAMPs), or damage associated molecular patterns (DAMPs) that are derived from doxorubicin‐induced damaged or dying cells.^[^ ^13^ ^]^ TLRs play a central role in intestinal homeostasis and dysregulated TLR signaling is associated with the pathogenesis of many intestinal inflammatory diseases.^[^ ^14^ ^]^ In doxorubicin‐induced intestinal mucositis, TLR2 and TLR9 play a central role as the absence of TLR2 and TLR9 in mice reduced doxorubicin‐induced peritoneal inflammation^[^ ^11^ ^]^ and intestinal inflammation.^[^ ^7^ ^]^ Inhibition of either TLR2 or TLR9 may therefore be an effective strategy to prevent the development of doxorubicin‐induced intestinal mucositis.

Previously it was demonstrated that the dietary fiber pectin inhibits TLR2^[^ ^15^, ^16^ ^]^ and is effective in limiting the development of mucositis in a microbiota‐independent manner.^[^ ^16^ ^]^ These effects of pectin on TLR2 were dependent on specific structural characteristics of pectins. Because of the conditions used commercially to isolate pectins, the isolated pectins are mostly homogalacturonans. However, in the unextracted cell walls of fruits and vegetables, the pectins contain additional domains including rhamogalacturonan I, rhamnogalacturonan II, and xylogalacturonan.^[^ ^17^ ^]^ Homogalacturonan pectins consist mainly of α (1‐4)‐linked galacturonic acid (GalA) residues. These GalA residues within the galacturonan backbone can be methyl esterified, as quantified by the degree of methyl‐esterification (DM). These methyl‐esters can be distributed in different patterns over the GalA backbone of the pectins, as expressed by the degree of blockiness (DB) of a pectin. Pectins with a low DB have a more random distribution of methyl‐esters over the GalA backbone, whereas pectins with a high DB have a more blockwise distribution of methyl‐esterified GalA residues and consequently also a more blockwise distribution of the non‐esterified GalA residues.^[^ ^18^ ^]^

Pectins with a low DM have stronger TLR2‐1 inhibiting properties than pectins with a high DM^[^ ^16^ ^]^ and low DM pectins were therefore very efficient in preventing the development of doxorubicin‐induced intestinal mucositis.^[^ ^16^ ^]^ In addition to these DM‐dependent effects, a recent study also showed that both the DM and the DB strongly impact the inhibition of TLR2‐1.^[^ ^19^ ^]^ Low DM pectins (DM19) and an intermediate DM pectin (DM46) with a high DB inhibited TLR2‐1 strongly, whereas intermediate DM pectin with low DB and high DM pectins (DM86) did not inhibit TLR2. Thus, the blockwise distribution of non‐esterified GalA in low DM pectins and the intermediate DM pectins play an important role in the anti‐inflammatory properties via TLR2‐1 signaling.^[^ ^19^ ^]^ How DM and DB of pectins contribute to the anti‐inflammatory effect against the development of doxorubicin‐induced intestinal mucositis is unknown.

We hypothesized that the number and distribution of methyl‐esters in pectin determine the anti‐inflammatory efficacy of pectins on doxorubicin‐induced intestinal mucositis. Four structurally different pectins, a low DM pectin with low DB, a low DM pectin with high DB, an intermediate DM pectin with low DB, and an intermediate DM pectin with a high DB were tested for inhibitory effects on murine TLR2 (mTLR2). Next, the anti‐inflammatory effect of these pectin structures in mice with doxorubicin‐induced intestinal mucositis was investigated by determining intestinal histology, intestinal apoptosis, intestinal barrier function, and peritoneal inflammation.

## 2 Results

### 2.1 Pectin Characterization

In the current study, we addressed the impact of DM and DB on mTLR2 signaling. To this end, four pectins were selected. These pectins were homogalacturonan pectins that did not differ in molecular weight or sugar composition (**Table** **1**). The pectins differed in DM and were grouped into two levels of similar DM: low DM pectins with a DM of 19% (DM18 and DM19) and intermediate DM pectins with a DM of 46% (DM43 and DM49). The pectins were also selected for their difference in DB but similar DM. Each DM group contained a pectin with a lower DB (DM18, DM49) and a pectin with a higher DB (DM19 and DM43). The DM18 and DM49 pectins were the pectins with a lower DB of 86 and 33, respectively, and the DM19 and DM43 were the high DB pectins that had a DB of 94 and 60, respectively.

**Table 1:** Structural characteristics of the pectins

Pectin	Origin	DB [%]^a)^	Mw [kDa]	Sugar composition [mol%]^a)^					Carbohydrate content [%]^a)^
				Rha	Ara	Gal	Glc	UA	
DM18	Lemon	86	78	1	0	2	0	97	62
DM19	Lemon	94	75	1	1	3	0	95	63
DM43	Lemon	60	79	0	0	0	0	99	77
DM49	Lemon	33	114	0	1	2	0	96	73

Pectins were characterized for the degree of methyl‐esterification (DM), degree of blockiness (DB), molecular weight (Mw), rhamnose (Rha), arabinose (Ara), galactose (Gal), glucose (Glc), and uronic acid (UA).^[^ ^19^ ^]^

^a)^ Values are the average of two replicates. Absolute deviations were always <3%.

### 2.2 Pectins Inhibit mTLR2 in a Dose and Structure‐Dependent Manner

**Figure 1:**
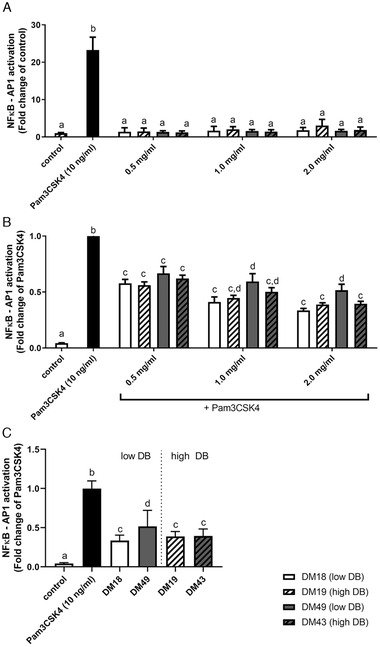
Murine TLR2 activation and inhibition by pectins. A) TLR2 activation and B) inhibition of TLR2 activation of mTLR2 by DM 18 (low DB) pectin, DM 19 (high DB) pectin, DM 49 (low DB) pectin, DM 43 (high DB) 0.5, 1.0, and 2.0 mg mL^−1^, and C) TLR2 inhibition by low DB and high DB pectins at 2.0 mg mL^−1^. Data is represented as mean ± SD (*n* = 15). Statistical differences (*p* < 0.05) were quantified using one‐way ANOVA with Tukey's post‐hoc test. ^a–d^ indicate statistical differences for each pectin concentration and were compared to control and Pam3CSK4.

First, the impact of DM and DB of pectin on mTLR2 stimulation or inhibition was investigated by testing the DM18 (low DB), DM19 (high DB), DM49 (low DB), DM43 (high DB) pectins on TLR2 activation or inhibition of Pam3CSK4‐induced TLR2 activation. As shown in **Figure** **1**, none of the pectins activated TLR2, but they had the ability to inhibit Pam3CSK4‐induced TLR2 activation. The effects of pectins on TLR2 inhibition were dose and structure dependent (Figure 1B). At 0.5 mg mL^−1^, TLR2 was inhibited by DM18 (low DB) with 42.2% (*p* < 0.05), DM19 (high DB) with 43.8% (*p* < 0.05), DM49 (low DB) with 32.3% (*p* < 0.05), and DM43 (high DB) with 38.0% (*p* < 0.05). Increasing the concentration of pectin gradually enhanced the inhibitory effect of the pectins. To better illustrate structural‐dependent effects of pectins on TLR2 inhibition, the inhibition of TLR2 with pectins at 2 mg mL^−1^ is shown in Figure 1C. Both low DM pectins with DM18 (and low DB) and DM19 (high DB) inhibited TLR2 at a similar strong level, independently of the DB. DM18 (low DB) inhibited TLR2 with 66.5% (*p* < 0.05) and DM19 (high DB) inhibited TLR2 with 61.3% (*p* < 0.05). The effect of DB was however more visible by pectins with an intermediate DM (DM49 and DM43). The DM43 (high DB) pectin inhibited TLR2 with 60.5% (*p* < 0.05) and significantly stronger than the DM49 (low DB) pectin that inhibited TLR2 with 48.3% (*p* < 0.05). The DM43 (high DB) pectin inhibited mTLR2 at a similar strong level as the low DM pectins (DM18 and DM19). These studies suggest that pectins with a blockwise distribution of non‐esterified GalA residues as found in both low DM pectins (DM18 and DM19) or in intermediate DM pectins (DM43) strongly inhibit mTLR2, whereas pectins with a random distribution of non‐esterified GalA residues in intermediate DM pectins (DM49) does inhibit mTLR2 less efficient.

### 2.3 Pectins Reduce Doxorubicin‐Induced Epithelial Apoptosis in a Structure‐Dependent Manner

**Figure 2:**
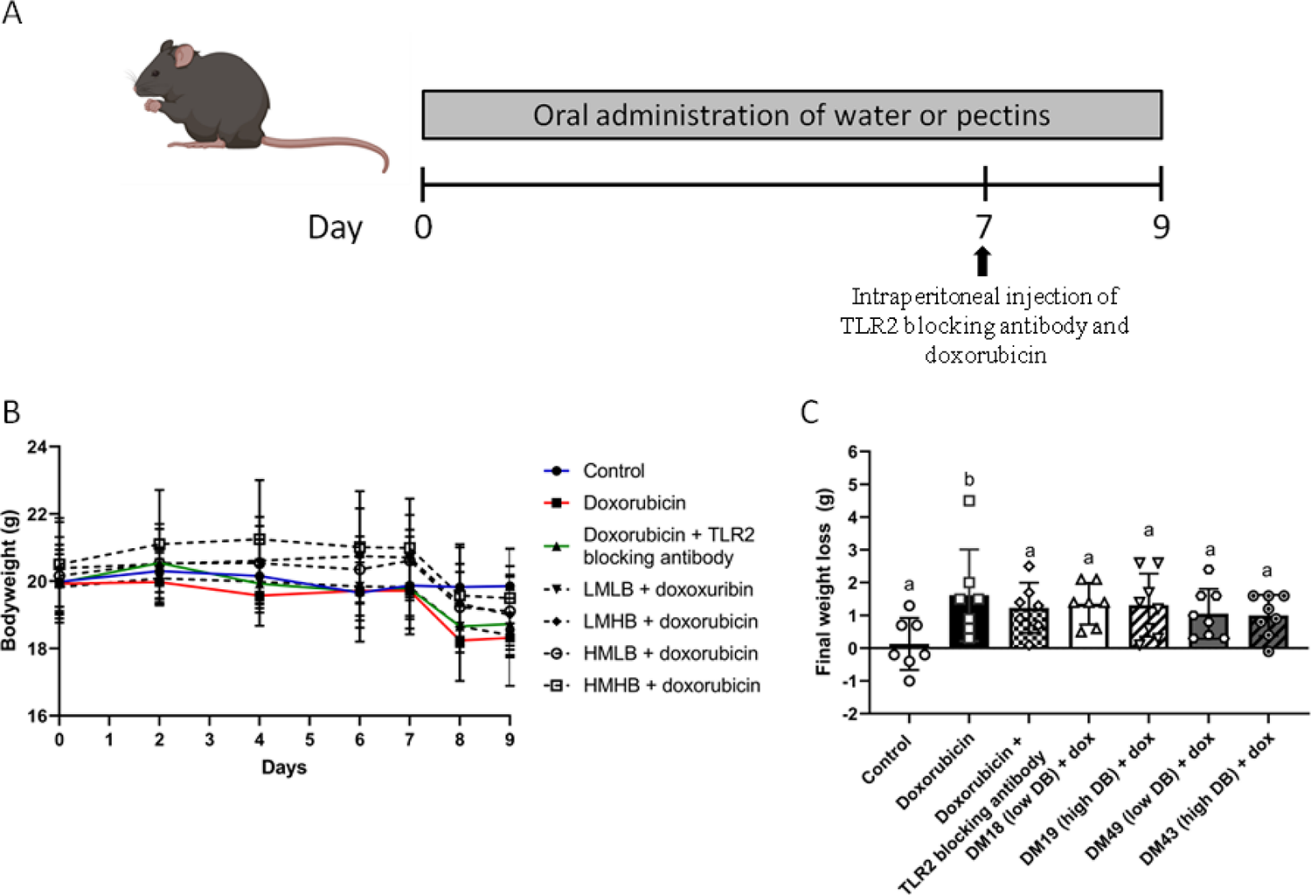
Study timeline and bodyweight development in the pectins and TLR2 treated doxorubicin treated mice. A) Mice received pectins or water via oral administration for a period of 9 days. On day 7, TLR2 blocking antibody and doxorubicin were intraperitoneally injected and mucositis was induced. Mice were sacrificed on day 9. B) Bodyweight was measured of control (*n* = 7), doxorubicin (dox; *n* = 7), doxorubicin + TLR2 blocking antibody (*n* = 7), doxorubicin + DM18 pectin (low DB; *n* = 7), doxorubicin + DM19 (high DB; *n* = 8), doxorubicin + DM49 (low DB; *n* = 8), and doxorubicin + DM43 (high DB; *n* = 8) treated mice to follow changes after pectin and doxorubicin treatment. C) Final weight loss compared to day 0 was also determined. Data is represented as mean ± SD. ^a–d^ indicate statistical differences (*p* < 0.05) between doxorubicin and other experimental groups as quantified with one‐way ANOVA test and Tukey's post‐hoc test.

Since pectins are known to inhibit TLR2 in a structure‐dependent manner, the protective effect of the different pectin structures on the development of TLR2‐dependent doxorubicin‐induced intestinal mucositis was investigated (**Figure** **2**). Mice received one of the four pectins during the whole experiment. After 7 days of pectin administration, doxorubicin‐induced intestinal mucositis was induced. Mice treated with TLR2 antibodies served as controls (Figure 2A). Doxorubicin‐induced a significant weight loss of 1.48 g (*p* < 0.05) in control mice treated with doxorubicin only (Figure 2B), but doxorubicin did not induce a significant change in bodyweight in mice treated with the different pectins or with TLR2 blocking antibody while a loss of bodyweight was observed in mice treated with doxorubicin only.

**Figure 3:**
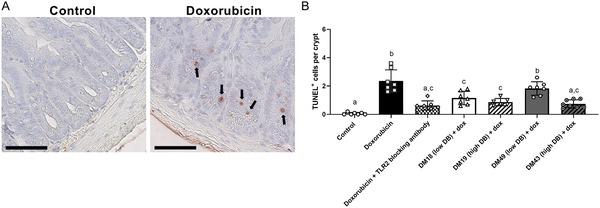
Effects of pectins on epithelial apoptosis. A) TUNEL^+^ staining of ileal sections was performed to determine apoptotic epithelial cells in crypts as is depicted for control and doxorubicin treated mice. B) The number of apoptotic cells per crypt was determined from mice. Data is represented as mean ± SD. ^a–d^ indicate statistical differences (*p* < 0.05) between doxorubicin and other experimental groups as quantified with one‐way ANOVA test and Tukey's post‐hoc test. Scale bar = 60 µm.

As doxorubicin rapidly induces apoptosis in a TLR2‐dependent manner,^[^ ^7^ ^]^ the impact of the different pectin structures on epithelial apoptosis was investigated by measuring TUNEL^+^ apoptotic cells in the crypt of the ileum. As shown in **Figure** **3**, the number of doxorubicin‐induced TUNEL^+^ cells was lower in crypts of pectin treated mice compared to mice treated with doxorubicin only. This protective effect of pectins on doxorubicin‐induced apoptosis was stronger with pectin structures that strongly inhibited TLR2. The strong TLR2 inhibiting pectins DM18 (low DB), DM19 (high DB), and DM43 (high DB) reduced apoptosis with 50.9% (*p* < 0.05), 63.2% (*p* < 0.05), and 68.7% (*p* < 0.05), respectively, and stronger than the weak TLR2 inhibiting pectin DM49 (low DB) that reduced apoptosis with only 22.4%. Additionally, mice treated with the stronger TLR2 inhibiting pectins significantly differed in the number of apoptotic cells from mice treated the weaker TLR2 inhibiting DM49 (low DB pectin). Compared to the DM49 (low DB) pectin treated mice, the number of apoptotic cells in the crypt were 37.6% (*p* < 0.05) lower in the DM18 (low DB) pectin treated mice, 52.9% (*p* < 0.05) lower than the DM19 (high DB) pectin treated mice, and 59.6% (*p *< 0.05) lower than the DM43 (high DB) pectin treated mice. Since doxorubicin‐induced epithelial apoptosis is prevented by pectins with strong TLR2 inhibiting properties and it is prevented at a similar level as the TLR2 blocking antibody, these findings suggest that these pectins prevent doxorubicin‐induced epithelial apoptosis by inhibiting TLR2.

### 2.4 Pectins Protect against Doxorubicin‐Induced Intestinal Damage Independently of TLR2 Inhibition

**Figure 4:**
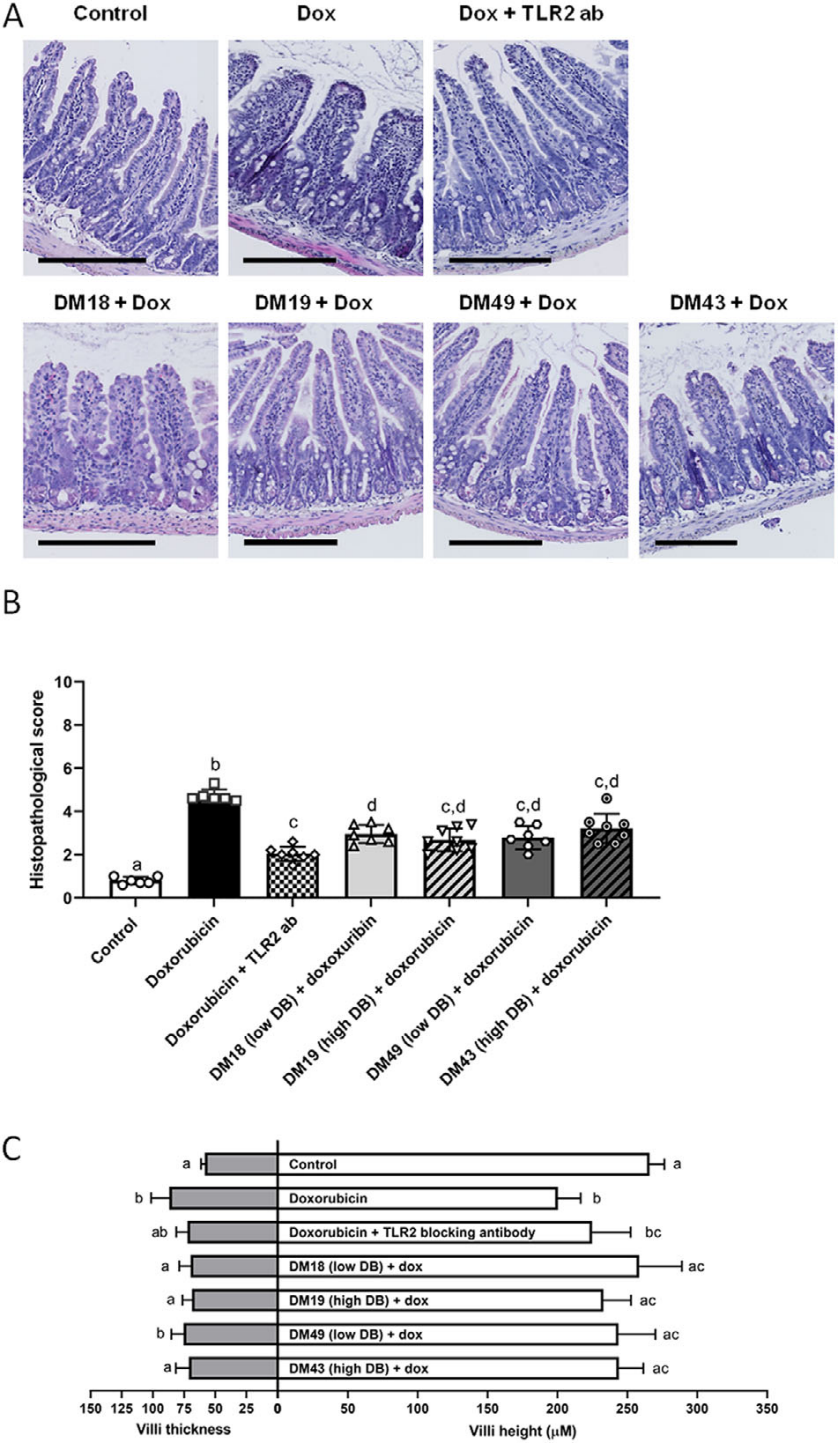
Protective effects of pectins against doxorubicin‐induced intestinal damage. A) H&E stained ileal segments were used to measure B) histological score, C) villus length and thickness from mice. Data is represented as mean ± SD. ^a–d^ indicate statistical differences (*p* < 0.05) between doxorubicin and other experimental groups as quantified with one‐way ANOVA test and Tukey's post‐hoc test. Scale bar = 200 µm.

Next, the histopathological score, villus degeneration, and villus thickening were examined to determine the protective effect of the different pectin structures on doxorubicin‐induced intestinal inflammation (**Figure** **4**). Pectin treated mice showed a significant lower histopathological score (2.95 for DM18 (low DB) (*p* < 0.05); 2.68 for DM19 (high DB) (*p* < 0.05); 2.79 for DM49 (low DB) (*p* < 0.05), and 3.21 for DM43 (high DB) (*p* < 0.05)) than mice treated with doxorubicin only (4.72 for doxorubicin only) (Figure 4B). Pectins also prevented doxorubicin‐induced villus degeneration with 88.6% (*p* < 0.05) for DM18 (low DB) pectin, 49.6% (*p* < 0.05) for DM19 (high DB) pectin, 66.2% (*p* < 0.05) for DM49 (low DB) pectin, and 66.6% (*p* < 0.05) for DM43 (high DB) pectin compared to doxorubicin treated only (Figure 4C). Furthermore, the DM18 (low DB) pectin prevented villus thickening with 60.0% (*p* < 0.05), the DM19 (high DB) pectin with 63.6% (*p* < 0.05), and the DM43 (high DB) pectin with 55.2% (*p* < 0.05; Figure 4C). The DM49 (low DB) pectin did not protect villus thickening significantly. Together, these findings show that all pectins prevented the development of doxorubicin‐induced intestinal mucositis independently of their TLR2‐inhibitory properties.

### 2.5 Pectins Limit Doxorubicin‐Induced Peritoneal Inflammation

Doxorubicin‐induced peritoneal inflammation is accompanied by neutrophil influx and increasing cytokine and chemokine production in the peritoneum.^[^ ^11^ ^]^ The influence of the different pectin structures on peritoneal inflammation was, therefore, investigated by measuring peritoneal neutrophil influx, Gro‐α, MCP‐1 chemokine levels, and TNF‐α, IL‐6, and IL‐10 cytokine levels.

**Figure 5:**
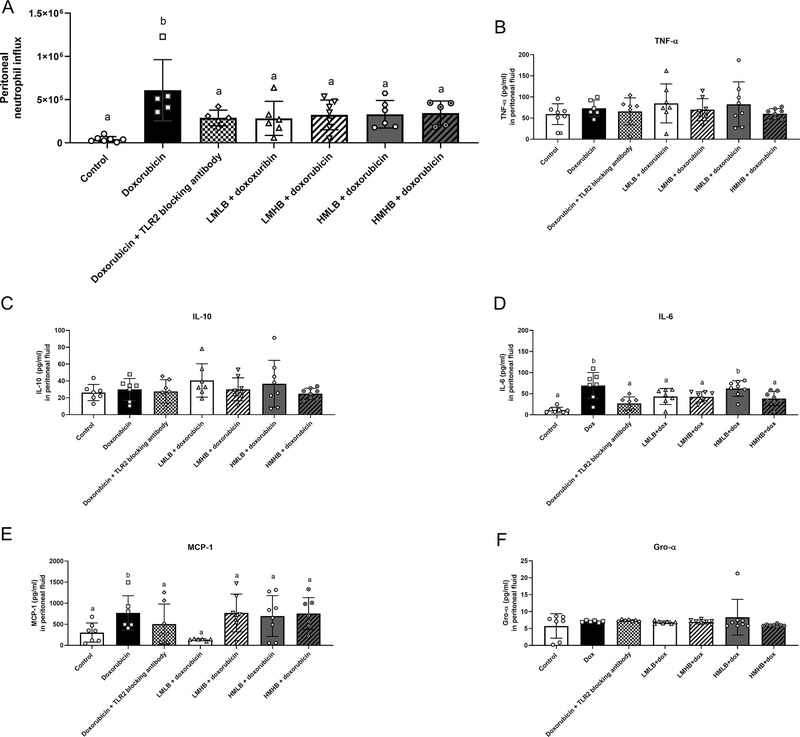
Impact of pectins on doxorubicin‐induced peritoneal inflammation. A) Neutrophil infiltration and levels of cytokines B) TNF‐α, C) IL‐10, D) IL‐6, E) MCP‐1, and F) Gro‐α were measured from the peritoneal fluid of mice. Data is represented as mean ± SD. ^a,b^ indicate statistical differences (*p* < 0.05) between doxorubicin and other experimental groups as quantified with one‐way ANOVA test and Dunnett's post‐hoc test.

All pectins limited the doxorubicin‐induced neutrophil influx in the peritoneal cavity (**Figure** **5**A) with 53.4% (*p* < 0.05) for DM18 (low DB pectin), 46.8% (*p* < 0.05) for DM19 (high DB) pectin, 45.7% (*p* < 0.05) for DM49 (low DB) pectin, and 43.3% (*p* < 0.05) for DM43 (high DB pectin). In addition, the pectins limited the doxorubicin‐induced neutrophil influx at a similar level as the TLR2 blocking antibody (52.6% (*p* < 0.05)).

The different pectins also lowered IL‐6 and MCP‐1 levels in the peritoneum (Figure 5). MCP‐1 was only significantly inhibited by the DM18 (low DB) pectin with 82.3% (*p* < 0.05), whereas the other pectins did not inhibit MCP‐1 signaling (Figure 5E). Peritoneal IL‐6 levels were reduced with 37.7% (*p* < 0.05) after treatment with DM18 (low DB) pectin, with 38.8% after treatment with DM19 (high DB) pectin (*p* < 0.05), and with 44.5% for DM43 (high DB) pectin (*p* < 0.05) treated mice and not in DM49 (low DB) pectin (10.4%) treated mice. The inhibition of IL‐6 production by these pectins did not differ significantly from the TLR2 blocking antibody (Figure 5D). Thus, all pectin structures prevent peritoneal inflammation by preventing a doxorubicin‐induced neutrophil influx. Effects on cytokines were pectin structure‐dependent as DM18 (low DB) pectin inhibited MCP‐1 production and DM18 (low DB), DM19 (high DB), and DM43 (high DB) pectins inhibited IL‐6 production in the peritoneum.

## 3 Discussion

Intestinal mucositis is a serious complication of chemotherapeutic agents that may prevent the completion of the anti‐cancer treatment.^[^ ^3^, ^4^ ^]^ Therapeutic strategies with anti‐inflammatory properties are therefore urgently needed to limit the development of intestinal mucositis. Pectin was found as anti‐inflammatory compound to prevent the development doxorubicin‐induced intestinal mucositis through direct inhibition of TLR2 and in a microbiota‐independent manner.^[^ ^16^ ^]^ It is, however, unknown which specific structural characteristics of the dietary fiber pectin exerts the highest anti‐inflammatory effects and may be used as therapeutic agents for doxorubicin‐induced intestinal mucositis. Recently it was found that the DM and the DB as specific structural characteristics of pectins play an important role in inhibiting TLR2‐1.^[^ ^19^ ^]^ Therefore, we investigated which combination of DM and DB prevents the development of doxorubicin‐induced intestinal mucositis most efficiently. Here, we found that pectins with a low DM and intermediate DM pectins with a high DB strongly inhibited mTLR2, whereas pectin with an intermediate DM and low DB was less efficient in inhibiting TLR2. Through inhibiting TLR2 activation, pectins reduced in vivo doxorubicin‐induced apoptosis, intestinal damage, and peritoneal inflammation.

Pectins inhibited mTLR2 in a structure‐dependent manner. Both low DM pectins and intermediate DM pectins with a high DB inhibited TLR2‐1 strongly, whereas intermediate DM pectins with a low DB did only mildly inhibit TLR2‐1. This indicates that pectins with a more blockwise distribution of non‐esterified GalA residues (both low DM pectins and intermediate DM pectins with a high DB^[^ ^18^ ^]^) have a stronger impact on mTLR2‐1 signaling than pectins with a random distribution of non‐esterified GalA residues (intermediate DM pectin with a low DB^[^ ^18^ ^]^). This similar pattern of inhibition of pectin structures has been found on human TLR2‐1,^[^ ^19^ ^]^ suggesting that these pectins bind to conserved regions in the murine and human TLR2‐1 heterodimers. This suggestion is corroborated by Sahasrabudhe et al.,^[^ ^16^ ^]^ who found that pectins bind to TLR2 ectodomains, which are highly conserved regions between murine and humans.^[^ ^20^ ^]^ These findings suggest, therefore, that TLR2‐1 inhibition is established through the interaction between blockwise distributed non‐esterified GalA residues in pectins and the TLR2 ectodomain.

Sahasrabudhe et al.,^[^ ^16^ ^]^ also found that TLR2 inhibiting pectins reduced the levels of doxorubicin‐induced apoptosis. Our data added to this study by showing that the low DM pectins and the intermediate DM pectin with a high DB prevented the induction of doxorubicin‐induced apoptosis, whereas the intermediate DM pectin with a low DB did not. Additionally, the pectins prevented apoptosis at a similar level as the TLR2 blocking antibody, suggesting that the structure‐dependent inhibition of apoptosis by pectins is mediated through inhibition of TLR2. TLR2 plays an important role in apoptosis as it is induced after high activation of TLR2 and is subdued in TLR2 knockout mice with doxorubicin‐induced intestinal mucositis.^[^ ^7^, ^11^, ^21^ ^]^ After doxorubicin injection, TLR2 is most likely not activated by PAMPs because studies with germ‐free mice and antibiotic‐treated mice demonstrated that doxorubicin induces apoptosis in a microbiota‐independent manner.^[^ ^8^, ^22^ ^]^ It is more likely that TLR2 is activated by high levels of DAMPs since doxorubicin induces massive cell death that leads to the release of DAMPs.^[^ ^11^ ^]^ These findings suggest therefore that both low DM pectins and the intermediate DM pectin with a high DB strongly block high activation of TLR2 by the DAMPs, which results in a reduced level of epithelial apoptosis. The lower impact of the intermediate DM pectin with a low DB on apoptosis may be related to the weaker TLR2 inhibiting capacity of this pectin.^[^ ^19^ ^]^

Doxorubicin‐induced apoptosis increases intestinal permeability,^[^ ^9^ ^]^ which is followed by intestinal damage, such as villus degeneration and thickening.^[^ ^7^, ^8^ ^]^ Our data showed that pectin administration prevented doxorubicin‐induced intestinal damage, but not in a structure‐dependent manner as was found for doxorubicin‐induced apoptosis. Instead, the different pectin structures prevented the development of intestinal damage to a similar level. These contradicting findings may be explained by the barrier‐enhancing properties of pectins on the intestinal epithelium.^[^ ^23^, ^24^, ^25^ ^]^ Although the apoptosis of the epithelium may enhance intestinal permeability, pectins can stimulate epithelial integrity of undamaged epithelial cells, which leads to a similar level of intestinal permeability between the different pectin structures. Preserving intestinal permeability prevents the translocation of bacteria or bacterial products over the intestinal barrier, and limits the development of severe inflammation and damage of the intestinal mucosa induced by these molecules.^[^ ^8^, ^22^ ^]^ These findings suggest therefore that the different pectin structures preserved the intestinal permeability to a similar level that protected from the development of inflammation and intestinal damage.^[^ ^9^ ^]^

Next to the intestinal inflammation, doxorubicin also induces peritoneal inflammation that is a TLR2‐dependent inflammation characterized by a neutrophil influx and high levels of cytokines and chemokines.^[^ ^11^ ^]^ The current study shows that the different pectin structures inhibited the neutrophil influx at a similar level, but they had a structure‐dependent impact on MCP‐1 and IL‐6 production in the peritoneum. Inhibition of the neutrophil influx and IL‐6 production was at a similar level as the TLR2 blocking antibody, suggesting that the structural inhibition of TLR2 by pectins inhibited peritoneal inflammation by the pectins. This TLR2‐dependent effect is in line with a previous study demonstrating that high activation of TLR2 stimulates IL‐6 secretion.^[^ ^26^ ^]^ Additionally, these findings also indicate that there is a link between intestinal inflammation and peritoneal inflammation as was also found in another study where peritoneal cytokine levels accurately represented intestinal inflammation in inflammatory bowel disease patients.^[^ ^27^ ^]^ This implies that orally administered pectins have inhibitory effects on TLR2 in the gastrointestinal tract and thereby prevent the development of peritoneal inflammation.

In the current study, we hypothesized that the number and distribution of methyl‐esters in pectin determine the anti‐inflammatory efficacy of pectins on doxorubicin‐induced intestinal mucositis. The current study demonstrates that pectins with a high number of non‐esterified GalA residues in pectins distributed in a blockwise fashion are most effective in preventing the development of TLR2‐dependent doxorubicin‐induced intestinal mucositis. Such pectins can prevent high TLR2 activation that prevents the induction of epithelial apoptosis. Consequently, pectins preserve intestinal barrier function and prevent intestinal and peritoneal inflammation. This knowledge is important for a better understanding of structural characteristics of pectins with anti‐inflammatory properties on doxorubicin‐induced intestinal mucositis and can be instrumental in the design of functional food applications. Consumers undergoing chemotherapeutic treatments with doxorubicin may benefit from consuming pectins with a blockwise distribution of non‐esterified GalA residues as they can reduce the inflammatory complications of the anti‐cancer treatment.^[^ ^7^, ^16^ ^]^

## 4 Experimental Section

### Pectins

Four commercially extracted pectins from lemons were obtained from CP Kelco (Copenhagen, Denmark). Molecular weight, monosaccharide content, the DM, and the DB were determined as previously described.^[^ ^19^ ^]^

### Cell Lines

To study the influence of pectins on mouse TLR2 signaling, HEK‐Blue mTLR2 cell line (Invivogen, Toulouse, France) was used. This reporter cell line expressed soluble embryonic alkaline phosphatase (SEAP). The SEAP reporter gene was placed under the control of a NF‐κB and an AP‐1 responsive promotor. Upon activation of mTLR2 by a specific agonist, high levels of intracellular NF‐κB would lead to secretion of SEAP that could be quantified by QUANTI‐Blue (Invivogen).^[^ ^16^, ^28^ ^]^ HEK‐Blue mTLR2 cells were cultured in DMEM culture media (Lonza, Basel Switzerland) containing 10% de‐complemented Fetal Calf Serum, 50 U mL^−1^ Penicillin (Sigma, St. Louis, MO, USA), 50 µg mL^−1^ Streptomycin (Sigma), and 100 µg mL^−1^ Normocin (Invivogen) according to the manufacturer's instructions.

### Reporter Cell Assay

To study whether pectins can activate or inhibit mTLR2, activation or inhibition assays were performed with pectins using HEK‐Blue cells expressing mTLR2 (Invivogen). HEK‐Blue mTLR2 cells were seeded in 96 well plates at 2.8 × 10^5^ cells mL^−1^ in 180 µL/well and were incubated overnight in DMEM medium. The next day, the DMEM medium was replaced by DMEM medium containing pectins in the concentrations of 0.5, 1, or 2 mg mL^−1^. Activation of mTLR2 was studied by treating the cells with the pectins for 24 h. Inhibition of the mTLR2 was studied by pre‐treating the cells with pectins for 1 h followed by addition of 20 µL of the Pam3CSK4 (10 ng mL^−1^; Invivogen). Culture medium was used as negative control and the TLR2 specific agonist Pam3CSK4 was used as positive control. After 24 h of incubation, media supernatant was mixed with QUANTI‐Blue (Invivogen) in a ratio of 1:10. After 1 h of incubation, NF‐κB activation was quantified at 650 nm using a Versa Max ELISA plate reader (Molecular Devices, Sunnyvale, CA, USA). Incubation steps were performed at 37 °C and 5% CO_2_. TLR activation data was represented as fold change compared to culture medium only. TLR inhibition data was represented as fold change compared to Pam3CSK4.

### Mice

C57BL/6 female mice (10 weeks old) were obtained from Janvier Laboratories, France. The experimental use of animals was approved by the Animal Ethical Committee of the University of Groningen (CCD application number AVD1050020171487). All mice were acclimatized for 1.5 week prior to the start of the experiment. Animals were cohoused with a total number of two mice in individual ventilated cages. Mice were fed ad libitum with RHB‐B (AB Diets, Woerden, The Netherlands). Initially, each group contained eight mice, but some animals were excluded before the study started due to suboptimal condition of the mice.

The four pectins were administered twice a day for 10 days via oral gavage in a volume of 250 µL of pectin solution (6 mg mL^−1^). This is a daily dose of 150 mg kg^−1^ for a mouse of 20 g (human equivalent dose of 12.2 mg kg^−1[^ ^29^ ^]^). Dose of pectins was previously determined.^[^ ^16^ ^]^ To determine the effective pectin dose for this study, pectins were administered at different doses to mice with doxorubicin‐induced mucositis (1, 2, and 3 mg day^−1^). A total of 3 mg day^−1^ was chosen as highest concentration, because the required 6 mg mL^−1^ was the highest pectin concentration for which a homogenous pectin solution could be obtained. It was observed that 3 mg day^−1^ of pectins was the pectin dose that showed most reduction in doxorubicin‐induced epithelial apoptosis and neutrophil influx.^[^ ^16^ ^]^ Mice received one of the four pectins. Control mice received water in a similar volume. Doxorubicin was dissolved in 0.9% sodium chloride solution. On day 8, intestinal mucositis was induced by intraperitoneal injection of 10 mg kg^−1^ doxorubicin. Control mice were included to confirm inhibition of intestinal mucositis by TLR2 blocking antibodies. These mice were intraperitoneally injected with human and murine specific TLR2 blocking antibody (10 mg kg^−1^ of clone T2.5, Invivogen) 1 h before doxorubicin injection. On day 10, mice were anesthetized with isoflurane/O_2_ and mice were sacrificed by cervical dislocation. Peritoneal fluid was collected by injection and aspiration of 2 mL PBS. Peritoneal fluid was stored on ice until cell counting. Ileal samples were collected for histological analysis.

### Histology

Ileum was cut in pieces of 0.3 mm and they were fixed in 4% paraformaldehyde in PBS and embedded in paraffin. Paraffin sections of 4 µm were cut and hematoxylin and eosin (H&E) staining was performed on the slides. The ileum sections were also stained for apoptosis using the TUNEL assay. The TUNEL assay was performed according to manufacturer's instructions of the ApopTag Peroxidase In Situ Apoptosis Detection Kit (Merck Millipore, Billerica, MA, USA). As peroxidase substrate, 3‐amino‐9‐ethylcarbazole (AEC) (5% AEC stock; 95% 0.05 M acetate buffer pH 4.9; 0.1% of 30% v/v H_2_O_2_ (Merck)) was used and incubated for 10 min. Hematoxylin was used as counterstain. The stained slides were scanned at a magnification of 40× using a Hamamatsu slide scanner (Hamamatsu Photonics, Hamamatsu, Japan). Histopathological scoring (ranging from 0 to 12) was performed by assessing epithelial‐, villus‐, and crypt damage, and stroma retraction on H&E stained slides by two individuals as previously described.^[^ ^7^ ^]^ Villus height and villus thickness were determined from 10 consecutive villi structures of three different ileum segments. The villus height was measured from the base of the villus to the top. Villus thickness was measured at the base of a villus. Apoptotic cells were measured in 10 sequential crypts in the ileum per mouse.

### Neutrophil Count and Cytokine Levels in Peritoneal Fluid

The number of cells in the peritoneal lavage fluid was measured using a Z Series coulter counter (Beckman Coulter, Brea, CA, USA). After counting, cells were diluted in 5.0 × 10^5^ cells mL^−1^, and 100 µL was used to prepare cytospins. The remaining solution was spinned down at 3000 × *g* and stored at −80 °C for cytokine measurements. The cytospin slides were stained with Giemsa (Merck Millipore) for 30 min at room temperature. The stained slides were scanned at a magnification of 20× using a Hamamatsu slide scanner (Hamamatsu Photonics), and neutrophil influx in peritoneum was determined by counting the number of neutrophils within 150 immune cells.^[^ ^7^ ^]^ The total number of neutrophils was calculated using the total cell count of the peritoneal lavage fluid. Cytokines (TNF‐α, IL‐6 and IL‐10, Gro‐a, and MCP‐1) from the peritoneal fluid were determined with ELISA (R&D systems) according to manufacturer's instructions.

### Statistics

The results were analyzed using Graphpad Prism program (La Jolla, CA, USA). Normal distribution was confirmed using the Kolmogorov–Smirnov test. Statistical comparisons for reporter cell assays were tested with repeated measured one‐way ANOVA. Statistical comparison for histology, barrier function, neutrophil influx, and peritoneal cytokines were performed using one‐way ANOVA for analysis of parametrically distributed data. Non‐parametrically distributed data were log‐transformed and analyzed with one‐way ANOVA. Post‐testing was performed with Tukey's post‐hoc test to determine statistical differences between all experimental groups. For peritoneal neutrophil influx and peritoneal cytokine levels, Dunnett's post‐hoc test was used to measure statistical differences between mice treated with doxorubicin only (dox.) and other treatments.

## Conflict of Interest

The authors declare no conflict of interest.

## Data Availability

Research data are not shared.
